# Langerhans Cell Histiocytosis of the Clavicle: A Case Report and Review of the Literature

**DOI:** 10.1097/MD.0000000000000117

**Published:** 2014-10-24

**Authors:** Shaowu Wang, Weisheng Zhang, Shengbo Na, Lina Zhang, Zhijin Lang

**Affiliations:** Department of Radiology (SW, WZ, LZ, ZL), First Affiliated Hospital of Dalian Medical University; and Department of Radiology (SN), Second People’s Hospital of Dalian, Dalian, China.

## Abstract

We report a rare case of solitary Langerhans cell histiocytosis (LCH) involving the clavicle of an adult female. The patient was a 32-year-old female presenting with 1 month history of progressive pain, swelling, and tenderness in the region near the left sternoclavicular joint. Radiograph, computed tomography, and magnetic resonance imaging showed an osteolytic lesion in the clavicle with tumor extension and soft tissue edema. Surgical curettage of the lesion was performed, and the histopathologic diagnosis was LCH. Because of its rarity and possibly variable presentation, LCH should be included and considered in the differential diagnosis when we encounter a clavicle lesion.

## INTRODUCTION

Langerhans cell histiocytosis (LCH) is a rare disease of unknown etiology with an estimated annual prevalence of 1 case per 560,000 in adults^1,2^ and encompasses 3 disorders: eosinophilic granuloma (EG), Hand–Schuller–Christian syndrome, and Letterer–Siwe syndrome according to their clinical and pathologic features. One of the most frequent presenting imaging features in adults is skeletal involvement with lytic lesions,^1^ but clavicle lesions are extremely rare, especially in adults.^3,4^ Till now, almost no related articles report characteristic imaging of LCH in clavicle in adults. We report 1 female adult patient with solitary clavicle EG, with a literature review.

## CASE REPORT

**FIGURE 1:**
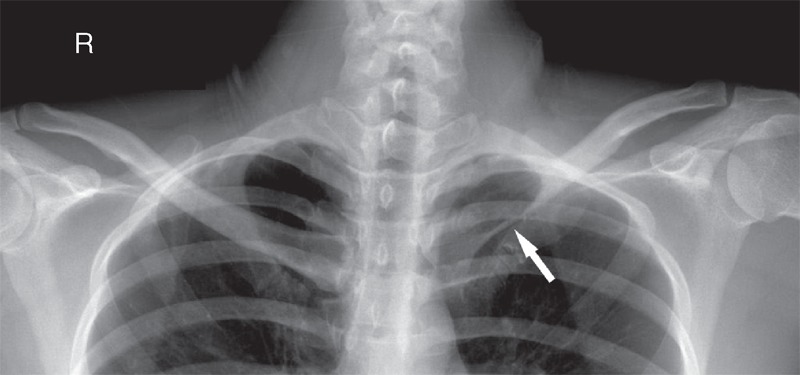
Posterior–anterior plain radiograph shows an ill-defined osteolytic lesion (arrow) without sclerotic margin in the medial part of left clavicle with soft-tissue swelling above the lesion.

**FIGURE 2:**
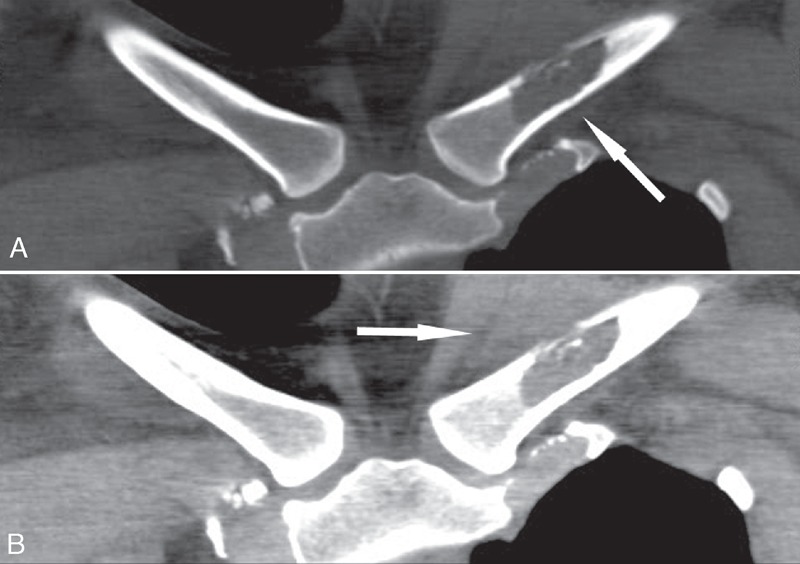
CT multiplanar reconstruction images show (A) an osteolytic lesion (arrow) with bone residual of the bone marrow and a disruption of the upper cortex in the diaphysis of the left clavicle and (B) surrounding tumor extension and soft tissue edema (arrow). CT = computed tomography.

**FIGURE 3:**
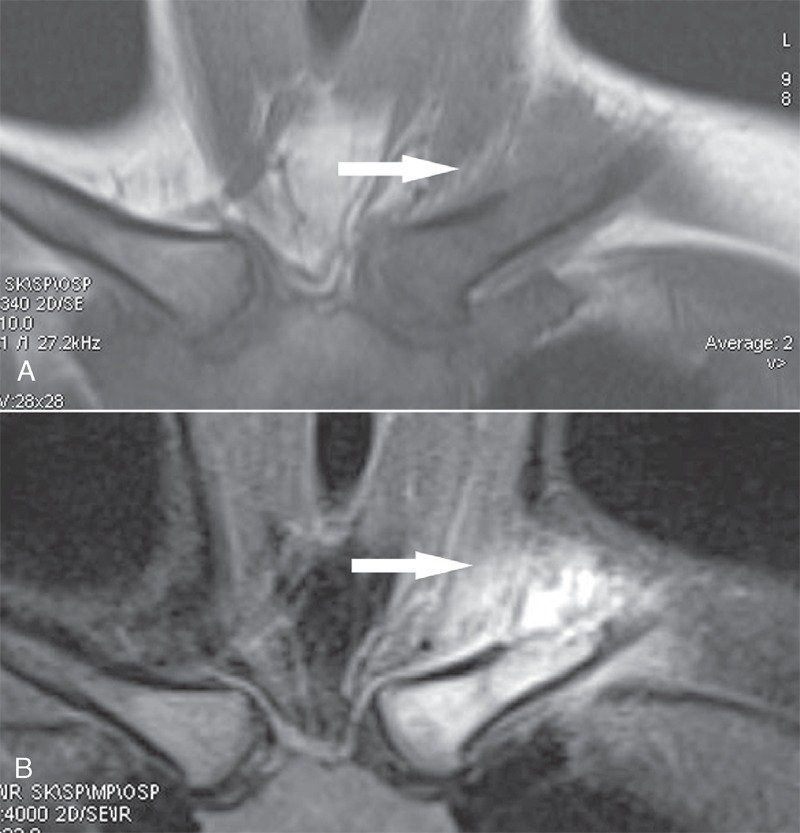
MRI shows the signal intensity of the tumor low in the left clavicle on (A) T1-weighted image and high on (B) T2-weighted image with tumor extension and soft-tissue edema (arrow). MRI = magnetic resonance imaging.

A 32-year-old female adult presented with 1 month history of progressive pain, swelling, and tenderness in the region near the left sternoclavicular joint. The patient denied fever, chills, night sweats, weight loss, and fatigue. There was no history of trauma. The posterior–anterior radiograph showed ill-defined osteolytic lesion without sclerotic margin in the left clavicle with soft tissue swelling above the lesion (Figure 1). Computed tomography (CT) multiplanar reconstruction images revealed the osteolytic lesion in the diaphysis of the left clavicle with surrounding swelling soft tissues (Figure 2). Moreover, there was no osteosclerosis and periosteal reaction. On magnetic resonance imaging (MRI), the lesion showed low signal intensity on T1-weighted images and high signal intensity on T2-weighted images with soft tissues extension above the clavicle (Figure 3). There were no other lesions in the systemic survey. All laboratory results were normal.

**FIGURE 4:**
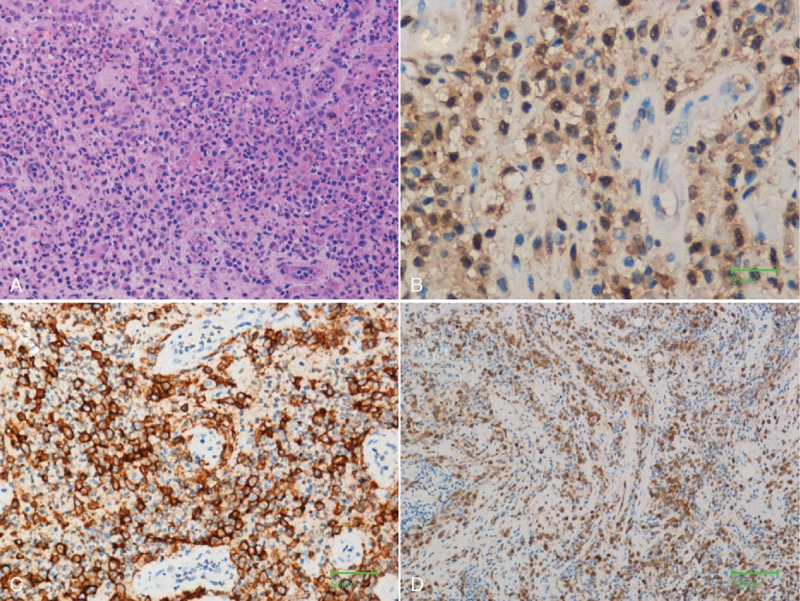
Histopathologic examination (100×) reveals (A) proliferation of histiocytes with an infiltration of eosinophils. Immunohistochemically, these histiocytes were positive for (B) S100 (+), (C) CD1a (++), and (D) Langerin (+).

**FIGURE 5:**
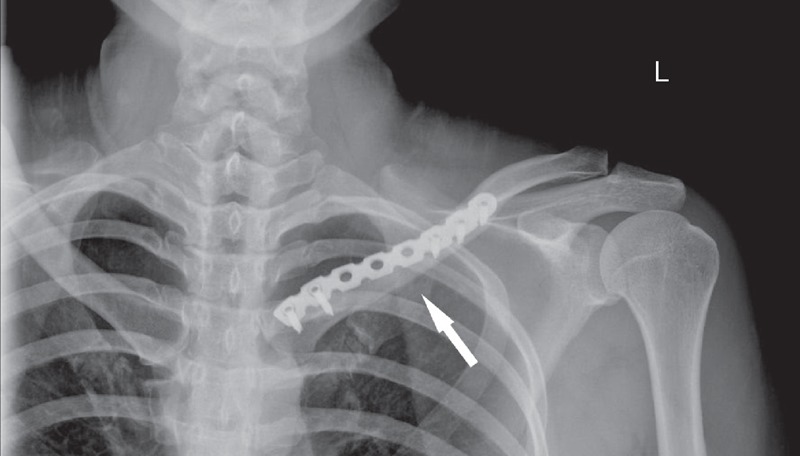
Internal steel plate fixation (arrow) is demonstrated after surgical curettage.

Surgical curettage was performed and histopathologic examination revealed a proliferation of histiocytes with an infiltration of eosinophils. Immunohistochemically, these histiocytes were positive for S100 (+), CD35 (+), CD1a (++), CD68 (+), VIM (++), Ki-67 (30%+), and Langerin (+) (Figure 4). A diagnosis of LCH was made. The patient was treated with internal steel plate fixation (Figure 5).

## DISCUSSION

LCH is an abnormal proliferation of tissue macrophages called Langerhans cells in 1 or more organs, including skin, lymph nodes, lung, liver, spleen, bone, and bone marrow. Patient age ranges from 5 to 15 years in about 90% of the cases with a slight male predominance.^5,6^ LCH accounts for <1% of tumor-like lesions of bone^7^ and the most frequent site is the skull and jaw, followed in decreasing order of frequency by long tubular bones, pelvis, ribs, spine, scapula, and clavicle.^4,8^ Clavicle lesions are extremely rare.^3,4,9,10^

Our patient was a female adult with a solitary lesion in the left clavicle. The plain radiograph showed an osteolytic lesion of the clavicle. CT demonstrated the proximal part extension of the lesion with bone residual and without sclerotic margin confirming disruption of the cortex and extension of lytic lesion. MRI showed low signal intensity on T1-weighted images and high signal intensity on T2-weighted images. A wide variety of bone lesions may mimic EG. Tumors and tumorous lesions of the clavicle should be excluded, such as Ewing sarcoma, aneurysmal bone cyst, desmoid tumor, myeloma, plasmacytoma, chondrosarcoma, and osteomyelitis.^11–14^ Ewing sarcoma and plasmacytoma often show permeative bone lesion with large soft tissue mass. Aneurysmal bone cyst can be geographical with sclerotic margin whereas myeloma can be geographical with ill-defined margin. Desmoid tumor is often with spherical bony outgrowth, no soft tissue mass, and no intraosseous extension. Chondrosarcoma may show huge soft tissue mass with permeative lesion.^11,13^ Osteomyelitis should be included for differential diagnosis and usually shows ill-defined bone destruction and sclerosis, expansion, and lamellated subperiosteal new bone formation.^12,14^ Although radiologic characteristics of the bony lesion indicated a diagnosis of LCH in the current case, we should stress that a diagnosis of LCH can only be finally made on histopathologic findings of a biopsy. An arrangement of histiocytosis in loose mesh-works or clusters and immunoreactivity for S-100 and CD1a antigens are helpful for the diagnosis of LCH.^6,15^ Because of its rarity and possibly variable presentation, the diagnosis of LCH may be overlooked or neglected. When we encounter a clavicle lesion, it might be difficult to remember the possibility of LCH.

Treatment of LCH depends on the extent of the disease. Various forms of treatment for a solitary lytic lesion affecting bones have been attempted, which include curettage, local steroid injection, radiotherapy, and chemotherapy alone or in combination. The results of treatment of solitary lesions are always satisfactory, although recurrence occurs in some patients (11%).^16^ In contrast, multifocal and multisystem types of LCH are generally treated with chemotherapy, in combination with other therapeutic modalities. In this patient, surgical curettage and internal steel-plate fixation were performed.
